# Supplementary material for: New Insights into Pediatric Kidney Transplant Rejection Biomarkers: Tissue, Plasma and Urine MicroRNAs Compared to Protocol Biopsy Histology
Source: Int J Mol Sci. 2024 Feb 5;25(3):1911. doi: 10.3390/ijms25031911 (PMC10856071; doi:10.3390/ijms25031911)
Supplement: Supplementary file 1 [file ijms-25-01911-s001.zip › ijms-2783953-supplementary.pdf]

sSEVs				
**Sample code**	**Banff score**	**Histological report**	**[]/mL**	**Diameter (nm)**
**CQA**	Banff 1	normal histology	1,92E+12	127,3 +/- 1,3 nm
**CQB**	Banff 1	normal histology	9,13E+11	105,9 +/- 1,2 nm
**CQC**	Banff 1	normal histology	1,45E+12	113,5 +/- 5,2 nm
**CQD**	Banff 1	normal histology	8,8E+11	145,1 +/- 5,7 nm
**CQE**	Banff 1	normal histology	4,94E+11	120,2 +/- 2,5 nm
**CQF**	Banff 1	normal histology	2,97E+12	142,4 +/- 8,7 nm
**CQG**	Banff 1	normal histology	7,02E+11	114,2 +/- 6,2 nm
**CQH**	Banff 1	normal histology	3,58E+12	111,7 +/- 0,7 nm
**CQI**	Banff 1	normal histology	6,16E+11	125,3 +/- 6,9 nm
**CQL**	Banff 1	normal histology	1,57E+12	132,2 +/- 1,3 nm
**CQM**	Banff 4	subclinical rejection	1,26E+12	135,0 +/-3,0 nm
**CQN**	Banff 3	subclinical rejection	2,41E+12	111,6 +/- 2,9 nm
**CQO**	Banff 3	subclinical rejection	4,1E+12	120,3 +/- 0,5 nm
**CQP**	Banff 3	subclinical rejection	1,31E+12	106,2 +/- 1,8 nm
**CQQ**	Banff 3	subclinical rejection	1,58E+12	134,0 +/- 1,3 nm
**CQR**	Banff 5	subclinical rejection	1,01E+12	118,3 +/- 5,3 nm
**CQS**	Banff 5	subclinical rejection	2,89E+12	124,3 +/- 2,4 nm
**CQT**	Banff 4	subclinical rejection	2,71E+12	111,5 +/- 0,8 nm
**CQU**	Banff 5	subclinical rejection	1,59+012	106,9 +/- 5,0 nm
**CQV**	Banff 5	subclinical rejection	9,19E+11	123,4 +/- 0,2 nm

Table S1. Serum sEVS (sSEVs). The table summarizes the first cohort's histological information, the particle concentration and the size of the sEVs purified from serum samples of patients with SCR (n=10) and normal histology (n=10) considered in this study.

UEVs				
**Sample code**	**Banff score**	**Histological report**	**[]/mL**	**Diameter (nm)**
**EDR**	Banff 1	normal histology	5,2E+11	188,5 +/- 11,5 nm
**EDT**	Banff 1	normal histology	6,34E+11	216,2 +/- 6,1 nm
**EDU**	Banff 1	normal histology	9,56E+11	180,8 +/- 2,6 nm
**EDQ**	Banff 1	normal histology	6,86E+11	183,2 +/- 1,9 nm
**EDV**	Banff 1	normal histology	5,64E+11	168,2 +/- 2,4 nm
**EEB**	Banff 2	subclinical rejection	6,7E+11	190,0 +/- 8,0 nm
**EEE**	Banff 3	subclinical rejection	9,56E+11	194,4 +/- 8,0 nm
**EEC**	Banff 4	subclinical rejection	7,56E+11	181,2 +/- 15,1 nm
**EED**	Banff 4	subclinical rejection	6,14E+11	183,3 +/- 5,4 nm
**EEG**	Banff 3	subclinical rejection	2,79E+11	187,9 +/- 8,1 nm

Table S2. Urine sEVS (sUEVs). The table summarizes the second cohort's histological information, the particle concentration and the size of the sEVs purified from urine samples of patients with SCR (n=5) and normal histology (n=5) considered in this study.

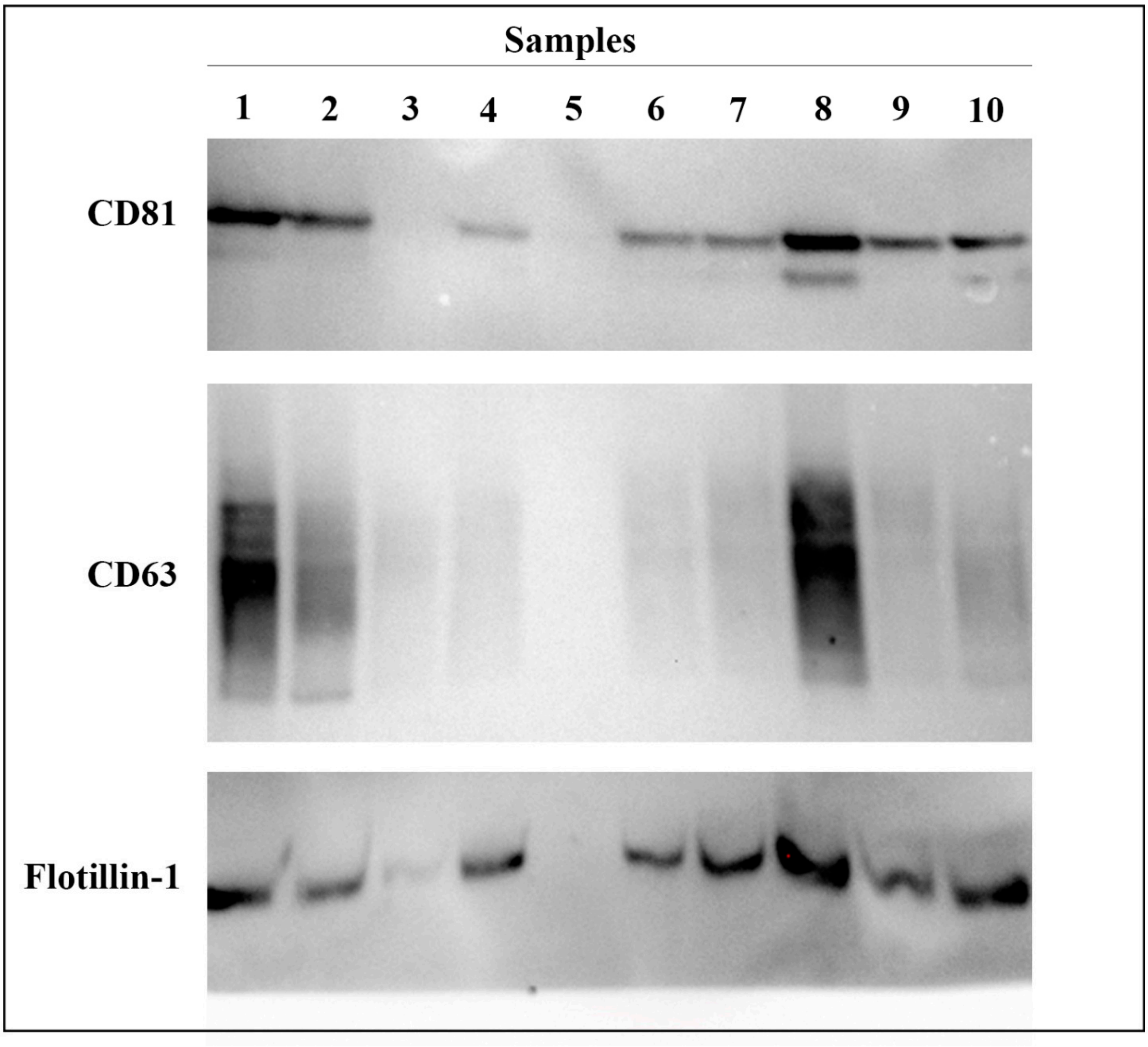

Figure S1. Western blot analysis. Blot of the two tetraspanins CD81 and CD63, and Flotillin-1 present in sUEVs. Samples 1 to 10 are EEB, EEE, EDV, EED, EEG, EDR, EDT, EDU, EDQ, EEC. Samples show a different markers expression. All samples were positive for at least one of the three markers. Flotillin-1 was the most present. The EDV sample shows a low markers expression, whereas the EEG sample seems to not have evidence of these proteins, probably due to its low concentration. This feature was commented on the Section 2.2. Extraction and Characterization of EVS.
